# Bioactive, Mineral and Antioxidative Properties of Gluten-Free Chicory Supplemented Snack: Impact of Processing Conditions

**DOI:** 10.3390/foods11223692

**Published:** 2022-11-17

**Authors:** Jelena Bokić, Jovana Kojić, Jelena Krulj, Lato Pezo, Vojislav Banjac, Vesna Tumbas Šaponjac, Vanja Travičić, Diego A. Moreno, Marija Bodroža-Solarov

**Affiliations:** 1 Institute of Food Technology, University of Novi Sad, Bul. Cara Lazara 1, 21000 Novi Sad, Serbia; 2 Institute of General and Physical Chemistry, University of Belgrade, Studentski Trg 12–16, 11000 Beograd, Serbia; 3 Faculty of Technology, University of Novi Sad, Bul. Cara Lazara 1, 21000 Novi Sad, Serbia; 4 Phytochemistry and Healthy Food Lab (LabFAS), Department of Food Science Technology, Centro de Edafología y Biología Aplicada del Segura (CEBAS-CSIC), University Campus of Espinardo—25, Murcia, E-30100 Murcia, Spain

**Keywords:** chicory root, bioactive compounds, antioxidative activity, gluten free snack, extrusion

## Abstract

This study aimed to investigate the impact of chicory root addition (20–40%) and extrusion conditions (moisture content from 16.3 to 22.5%, and screw speed from 500 to 900 rpm) on bioactive compounds content (inulin, sesquiterpene lactones, and polyphenols) of gluten-free rice snacks. Chicory root is considered a potential carrier of food bioactives, while extrusion may produce a wide range of functional snack products. The mineral profiles were determined in all of the obtained extrudates in terms of Na, K, Ca, Mg, Fe, Mn, Zn, and Cu contents, while antioxidative activity was established through reducing capacity, DPPH (2,2-diphenyl-1-picrylhydrazyl) and ABTS (2,2-azino-bis(3-ethylbenzothiazoline-6-sulfonic acid) tests. Chicory root addition contributed to the improvement of bioactive compounds and mineral contents, as well as antioxidative activities in all of the investigated extrudates in comparison to the pure-rice control sample. An increase in moisture content raised sesquiterpene lactones and minerals, while high screw speeds positively affected polyphenols content. The achieved results showed the important impact of the extrusion conditions on the investigated parameters and promoted chicory root as an attractive food ingredient in gluten-free snack products with high bioactive value.

## 1. Introduction

Human nutrition habits have recently been focused on innovative healthy food. The development of gluten-free foods intended for people suffering from celiac disease, vegetarians, or people practicing a gluten-free diet based on rice, buckwheat, amaranth, and quinoa for some other reason is a technological challenge nowadays. Celiac disease is an autoimmune disorder that affects about 1% of the world’s population, but a strict gluten-free diet is practiced by about 10% of the population [1]. Celiac disease might be alleviated only by adhering to a gluten-free diet [2]. Among gluten-free materials, rice possesses some advantages: easy digestion, hypoallergenicity, mild taste, and colorlessness [3]. In addition to the gluten-free composition, rice is an important material in the production of extruded foods due to its high starch content. However, to improve the nutritional composition of rice extrudates, which are often enriched with protein or dietary fiber-rich raw materials [4]. Therefore, gluten-free snacks appear to be a promising basis for fortification. The roots and rhizomes of plants, representing rich sources of bioactive and nutritional valuable compounds, are attracting the interest of food technologists with the goal of developing novel functional products.

Chicory (*Cichorium intybus* L.) root is a rich source of inulin, oligofructose, polyphenols, and sesquiterpene lactones, observed as potential carriers of food functionality [5,6]. Inulin, as non-digestible dietary fiber, is a linear fructose polymer with β (2→1) glycosidic linkage, potentially causing antihyperglycemic and antidyslipidemic effects and improving bowel movements [7]. Chicory root flour causes different health effects than inulin alone, affecting cancer prevention, antibacterial and antiviral defense, hypoglycemic and hypolipidemic response, and antioxidant activity [6]. This statement may be related to the presence of other bioactive compounds in chicory root, such as sesquiterpene lactones (SLs). SLs, the bitter compounds from chicory, including dihydrolactucin, lactucin, 8-deoxylactucin, jacquinelin, dihydrolactucopicrin, and others, are C15 terpenoids that show primarily anti-inflammatory health benefits [8]. In addition, Perović et al. [5] highlighted chicory as a rich source of a variety of phenolic compounds managing antioxidative properties, including mono- and dicaffeoylquinic acids, chicoric acid, chlorogenic acid, caffeic acid, and others. Minerals are essential components of the human diet that serve as cofactors in the various enzyme-controlled reactions that enable the normal functioning of the human body [9]. Among selected pasture plants, such as dandelion and white clover, chicory root showed an abundant mineral content [10]. Product enrichment with whole chicory root flour instead of isolation and implementation of individual bioactive compounds may prevent the accumulation of by-products. Aiming to produce a rice-based snack with an improved bioactive profile, chicory root was chosen as an extrudate supplement.

Extrusion is flexible food technology that ensures improved digestibility and high sensory quality of final products [11]. A combination of high temperature and mechanical shear under pressure during extrusion cooking changes raw materials and modifies the functional properties, nutrient, and phytochemical composition of the food [12]. Several studies investigated the impact of the extrusion process on the nutritional composition, bioactive constituents, antioxidant potential, and the physicochemical and functional characteristics of rice-based extrudates [4,13]. Extrusion affected the investigated phytochemicals in different manners in the study of Arribas et al. (phenols increased while lectins and protease inhibitors were eliminated during extrusion) [4]. Dilrukshi et al. noted that extrusion also increased the oligosaccharides, while the insoluble fiber content was not significantly affected during the production of gluten-free extruded snacks fortified with cowpea and whey protein concentrate [13].

The objective of this study was to define the impact of the extrusion process variables (moisture content, screw speed, and chicory root flour addition) on the contents of bioactive compounds (inulin, sesquiterpene lactones, and polyphenols) and minerals, as well as the antioxidant activity of the obtained snacks. An artificial neural network (ANN) was employed to generate the dependence on the final product characteristics concerning the input extrusion process parameters.

## 2. Materials and Methods

The details of the raw materials’ characteristics, the preparations of the blends, the applied instruments, and the production steps of the rice-based extrudates enriched with chicory root are presented in a previously published paper [14]. Briefly, a twin-screw extruder, Bühler BTSK 30/28D (Bühler, Uzwil, Switzerland), was employed to produce novel gluten-free rice snacks with varying feed moisture (M, 16.3–22.5%), screw speed (SS, 500–900 rpm), and chicory root flour content (P, 20–40%), according to the central composite design (CCD). The rice-based extrudates enriched with chicory root flour were generated from five different blends (with 20, 24.1, 30, 35.9, or 40% chicory root), while the control sample (CS) was obtained from pure rice flour.

### 2.1. Inulin Determination

Then, 1 g of the ground sample was extracted with 10 mL of distilled water for 1 h in an agitation water bath (80 °C), which continued with 30 min of ultrasonication (ATU Ultrasonidos, Valencia, Spain). After centrifugation (10,000 rpm, 15 min; Eppendorf Centrifuge 5804R, Eppendorf, Wien, Austria), the supernatant was precipitated with four volumes of pure acetone (Avantor^®^, Radnor Township, PA, USA), overnight at 4 °C. The precipitates were redissolved in a water bath (80 °C) for 1 h, with an additional 30 min sonication. Appropriate dilutions of the samples were filtrated (0.45 µmØ pore size, regenerated cellulose from Agilent Technologies, Santa Clara, USA) prior to chromatographic analysis. The detection and quantification of inulin were determined by applying a high-performance liquid chromatographic method with the evaporative light-scattering detection (HPLC-ELSD) method developed and validated by Perović et al. [15]. This HPLC-ELSD analysis (16 min) has been performed by isocratic elution with water as a mobile phase and optimized detector parameters (the temperature of the evaporator was 80 °C, the temperature of the nebulizer was 80 °C, the gas flow rate was 1.3 standard liters per minute, and the detector gain was 1). The proposed method was carried out using a cation exchange Rezex™ RSO-Oligossacharide Ag+ (Phenomenex, Aschaffenburg, Germany) column (4%, 12 μm particle size, 200 × 10 mm).

### 2.2. Sesquiterpene Lactones (SLs) Content

The most represented SLs from chicory root: lactucin, lactucopicrin, di-hydrolactucin, and di-hydrolactucopicrin were extracted according to the procedure of Schmidt et al. [16]. The extraction was carried out by shaking with 95% Ethanol (Zorka Pharma-Hemija DOO, Šabac, Serbia) for 24 h at room temperature, with a ratio of sample:extraction agent = 1:10. The defatting of ethanolic extract with the n-heptane was carried out in a separatory funnel. The defatted ethanolic extract was further extracted with ethyl acetate to obtain a concentrated extract of sesquiterpene lactones. The evaporated extracts (under a stream of nitrogen) were dissolved in 2 mL of extractant (25:1:24 = methanol:formic acid:distilled water), placed in an ultrasonic bath for 10 minutes, filtered through 0.22 µm filters (PVDF, Millipore, Burlington, US), and subjected to chromatographic analysis.

The chromatographic analyses were carried out on a Luna C18 column (250 × 4.6 mm, 5 mm particle size; Phenomenex, Macclesfield, UK). Water/formic acid (99:1, *v/v*) and acetonitrile were used as the mobile phases A and B, respectively, with a flow rate of 1 mL/min. The linear gradient started at 0 min–5% B; 20 min–80% B, for separation; at 25 min–95% B, 30 min–95% B; wash; and back to initial 35 min–5% B, 40 min–5% B. The injection volume was 20 µL. Chromatograms were recorded at 254 and 320 nm.

The HPLC–DAD–ESI/MSn analyses were carried out using an Agilent HPLC 1200 system (Agilent Technologies, Waldbronn, Germany) coupled to a mass detector in series. The HPLC system consisted of a binary capillary pump (model G1376A), an autosampler (model G1377A), a degasser (model G1379B), a sample cooler (model G1330B), and a photodiode array detector (model G1315D) was controlled by ChemStation software (v.B.0103-SR2). The mass detector was a Bruker model UltraHCT (Bremen, Germany) ion trap spectrometer equipped with an electrospray ionization interface (ESI) and controlled by Bruker Daltonik Esquire software (v.6.1) (Bruker Daltonik GmbH, Bremen, Germany). The ionization conditions were 350 °C and 4 kV, for capillary temperature and voltage, respectively. The nebulizer pressure and nitrogen flow rate were 65.0 psi and 11 L/min, respectively. The full-scan mass covered the range of *m/z* from 100 to 1000. Collision-induced fragmentation experiments were performed in the ion trap using helium as the collision gas, with voltage ramping cycles from 0.3 to 2 V. The mass spectrometry data were acquired in the negative ionization. MS^n^ was carried out in the automatic mode on the more abundant fragment ion in MS^(n−1)^.

### 2.3. Mineral Elements

Mineral content analysis was performed on the Varian spectra AA 10 (Varian Techtron Pty Limited, Australia). Na^+^, K^+^, Ca^2+^, Mg^2+,^ Fe^3+^, Mn^2+,^ Zn^2+,^ and Cu^2+^ content in the extrudates and control samples were determined following the standard SRPS EN ISO 6869/2008 [17]. A mixture of air and acetylene gas was used in all experiments. Cathode lamps were used for radiation. The sensitive wavelengths for the detection of the investigated elements were: 330.3 nm for Na^+^, 404.4 nm for K^+^, 422.6 nm for Ca^2+^, 202.6 nm for Mg^2+^, 248.3 nm for Fe^3+^, 279.5 nm for Mn^2+^, 213.9 nm for Zn^2+^, and 324.7 nm for Cu^2+^.

### 2.4. Total Phenolic Content and Antioxidant Capacity

The sample (5 g) was poured with 12.5 mL of ethanol:water mixture (80:20, *v/v*) and extracted for 15 min in an ultrasonic bath at 40 °C, centrifuged (3000 rpm/10 min) (Centrifuga Tehtnica, Železniki, Slovenia), and the supernatant was evaporated to dryness in a stream of nitrogen at 40 °C on the Reacti-Therm I device (Thermo Fisher Scientific, Waltham, MA, USA). The dry residue was stored at −18 °C until analysis of the fraction of free polyphenolic compounds (Free PPs). The separated solid phase (precipitate) was used for determining the fraction of bound polyphenolic compounds (Bound PPs).

Bound PPs were released using alkaline hydrolysis with reflux (20 min). The precipitate was transferred to a flat-bottom flask and hydrolyzed using 50 mL of methanol, 5 mL of potassium hydroxide:water (1:1, *w/w*), and butyl-hydroxytoluene (BHT). The cooled hydrolysates were filtered through a Büchner funnel through qualitative filter paper (Whatman, Grade 4 Chr, Maidstone, Great Britain), and the filtrate was transferred to a separation funnel. The hydrolysate was initially neutralized with an HCl concentration of 6 mol/L, and protein precipitation was carried out with NaCl. The liquid–liquid extraction procedure was performed using a separatory funnel with 50 mL of the diethylether:ethylacetate (1:1, *v/v*). The water–methanol layer was extracted two more times, and the collected fractions were then evaporated on a rotary vacuum evaporator. The evaporated residue was dissolved using an ethanol:water mixture (80:20, *v/v*), after which the polyphenol content was determined on a Multiscan GO microtiter plate reader (Thermo Fisher Scientific Inc., Waltham, MA, USA).

#### 2.4.1. Free and Bound Polyphenols

Singleton and Rossi [18] described a procedure for determining the content of polyphenols based on their reaction with the Folin–Ciocalteu reagent, which was modified for 96-well microtiter plates [19].

#### 2.4.2. Antioxidant Capacity

The antioxidant capacity, expressed as mg Trolox equivalent (TE) per g of dry sample (mg TE/g), was determined by the 2,2-diphenyl-1-picrylhydrazyl method (DPPH) described by Gironés-Vilaplana et al. [20], 2,2′-azino-bis-3-ethylbenzothiazoline-6-sulphonic acid method (ABTS) adapted for microtiter plate [21], and reducing capacity (RC) method detailed by the Oyaizu [22]. All of the tests were carried out in triplicate, both for the free (DPPHF, ABTSF, RCF) and bound (DPPHB, ABTSB, RCB) polyphenolic fractions.

### 2.5. Artificial Neural Network

Three multi-layer perceptron (MLP) models, which have three layers (input, hidden, and output), were applied to model the ANN for the prediction of inulin, lactone, mineral, and polyphenol content and antioxidant activity as a function of moisture content, screw speed, and chicory root content. Before ANN computation, the database was normalized to improve the result accuracy of the ANN model. The normalization of the data was performed according to the min–max normalization criteria [23].

The Broyden–Fletcher–Goldfarb–Shanno (BFGS) algorithm was applied to solve unconstrained nonlinear problems during the optimization of the ANN models.

The exploratory data for ANN were randomly separated into training, cross-validation, and testing data (with 60%, 20%, and 20% of the experimental database, accordingly). A sequence of diverse topologies was employed, in which the number of hidden neurons ranged from 3 to 10, and the training process of the network was run in 100,000 repetitions using random weights and biased initial values. The optimization process was accomplished based on error minimization during the ANN validation cycle. The successful training was completed when the learning and the cross-validation curves approached zero [23,24,25,26].

Coefficients related to the hidden layer (weights and biases) were introduced into matrices *W*_1_ and *B*_1_. Similarly, coefficients related to the output layer were described in matrices *W*_2_ and *B*_2_. The neural network model (Y) can be represented using a matrix notation [23]:

(1) $$Y=f_{1}\left(W_{2}\cdot f_{2}\left(W_{1}\cdot X+B_{1}\right)+B_{2}\right)$$

where, *f*_1_ and *f*_2_ are transfer functions in the hidden and output layers, respectively, and *X* is the matrix of input variables.

ANN models and global sensitivity analysis of the obtained results were completed using Statistica 10.0^®^ software (StatSoft, Tulsa, OK, USA).

#### Local Sensitivity Analysis

Yoon’s local sensitivity formula for the developed ANN model was used to evaluate the relative influence of the input parameters on output variables based on the weight coefficients of the developed ANN models [27].

(2) $$RI_{ij}(\%)=\frac{\sum_{k=0}^{n}\left(w_{ik}\cdot w_{kj}\right)}{\sum_{i=0}^{m}\left|\sum_{k=0}^{n}\left(w_{ik}\cdot w_{kj}\right)\right|}\cdot 100\%\;$$

where: *w*—weight coefficient in ANN models; *i*—input variable; *j*—output variable; *k*—hidden neuron; *n*—number of hidden neurons; and *m*—number of inputs.

### 2.6. Statistical Analysis

The central composite design generated 20 snack formulations with six repetitions in the central point (samples 2, 4, 9, 14, 19, and 20). All of the experiments were performed in appropriate replications, and the results are presented as mean ± SD. The determination of the differences between the means and the samples was conducted using one-way ANOVA and Tukey’s multiple range test (*p* < 0.05). Statistical data processing was carried out using the statistical package XLSTAT 2020.5.1 (Addinsoft, New York, NY, USA).

## 3. Results and Discussion

### 3.1. Inulin Content

Up to now, the recommended daily intake of inulin has not been defined, and no toxic effect caused by high doses of inulin has been reported. Inulin can be used as a food ingredient and is most often labeled as “dietary fiber” on the packaging of the product, while mentioning the “bifidogenic effect” is also legal in several countries [28].

The inulin content in the analyzed samples varied from 3.29% to 10.10% (Table 1), while inulin was not detected in the control sample made from rice flour. Similarly, Radovanovic et al. reported an inulin content of 5.23–18.23% after the addition of Jerusalem artichoke (30–80%) into buckwheat extrudates [29].

The intake of 8–10 g of inulin per day may reduce the level of triglycerides, cholesterol, and LDL cholesterol [30]. Furthermore, consuming 5–15 g of inulin per day for several weeks has been shown to have prebiotic activity [31]. Considering these facts, it is notable from Table 1 that most of the gluten-free chicory-enriched extrudates designed within this study might cause some health effects.

**Figure 1:**
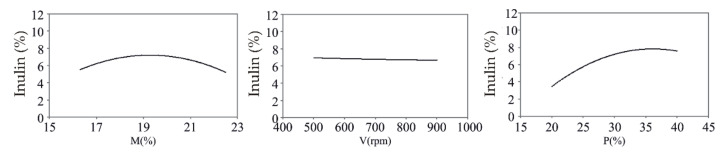
Influence of extrusion conditions (M—moisture content; V—screw speed; P—chicory root content) on inulin content.

The influence of M, V, and P were calculated using Yoon’s local sensitivity formula (Equation (1)), while the relative influence of these variables on the inulin content is shown in Figure 1.

The content of inulin decreases at higher moisture values (M > 19%, Figure 1), similar to the research of Ferreira et al. [32]. Namely, higher moisture content can encourage the dissolution of inulin, which makes it more exposed to shear forces and degradation during extrusion. Therefore, there is a noticeable decrease in the content of inulin when the moisture content is higher than 19% (Figure 1). Similar to obtained results, Sharma and Gujral [33] point out that a moisture content of up to 17% does not harm the content of dietary fibers, such as inulin.

The increase in screw speed showed a slightly decreased tendency for the inulin content (Figure 1), probably due to the intensified shear forces responsible for the degradation of inulin [34]. Similar conclusions were drawn by Tsokolar-Tsikopoulos et al. in a study examining the properties of expanded products with the added inulin [35].

The inulin content increased with the addition of chicory root (Figure 1), confirmed with a high positive correlation (r = 0.72, *p* < 0.05). This was expected, considering that chicory root is one of the richest sources of inulin [5,36]. Similarly, a significant increase in the inulin content (*p* < 0.05) was recorded in buckwheat-based extrudates with the addition of Jerusalem artichoke root rich in inulin [29].

### 3.2. Sesquiterpene Lactones (SLs)

Chicory sesquiterpene lactones have been reported to possess considerable biological activities and have been used in traditional medicines for centuries. Moreover, foods rich in SLs might be considered to be part of a healthy, balanced diet [37]. There is a noticeable lack of scientific studies that analyzed the content of SLs in food products, as well as the influence of processing conditions on the content of these bioactive compounds. Kulkarni et al. examined the influence of extrusion cooking on the bioavailability of the sesquiterpene lactone artemisinin, concluding that adjusting the pH (slightly acidic environment) can contribute to the preservation of this bioactive compound [38].

The presences of SLs in the extrudates were within the following ranges: 0.51–9.13 µg/g for lactucin, 0.56–5.52 µg/g for lactucopyrin, 0.49–12.39 µg/g for dihydrolactucin, and 0.00–4.07 µg/g for dihydorolactucopicrin (Table 1). Sample 16 contained the highest concentrations of all of the examined SLs (Table 1), which was expected considering that this sample had the highest proportion of chicory root (40%). The control sample did not contain any of the analyzed SLs.

**Figure 2:**
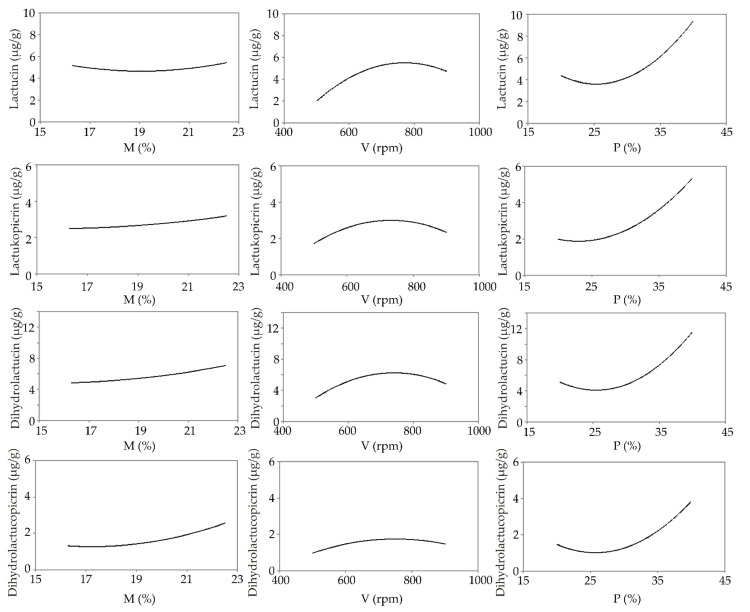
Influence of extrusion conditions (M—moisture content; V—screw speed; P—chicory root content) on sesquiterpene lactones content.

The effect of the M, V, and P variables was evaluated using Yoon’s local sensitivity formula, and the relative influence of these variables on the sesquiterpene lactone content is shown in Figure 2.

Increased moisture content had a positive effect on the content of all of the examined SLs (Figure 2), probably due to the facilitated release of SLs from the complexes. Similarly, moisture affected the solubility of artemisinin, a bioactive compound containing a sesquiterpene ring, thereby increasing its bioavailability [38]. Aberham et al. [39] examined the stability of sesquiterpene lactones (such as absinthe) in an aqueous environment, showing that the aqueous solutions of the lactone were stable for up to 6 months.

A screw speed up to 700 rpm caused an increase in SLs, while a further increase in V (above 700 rpm) negatively affected the content of the lactones (Figure 2). It is assumed that the higher screw speeds cause stronger shearing, frictional forces, and heating, whereby the partial or complete degradation of the analyzed lactones can occur. Screw speeds of 700 rpm and lower probably cause the release of lactones from the complexes, causing an increase in their bioavailability.

The addition of chicory root caused an increase in the content of all SLs, followed by high positive correlations (r = 0.65 to 0.75, *p* < 0.05). These results were expected due to the fact that chicory root is rich in SLs [40].

### 3.3. Total Phenolic Content and Antioxidant Capacity

Although selective and sensitive high-performance liquid chromatography provides reliable information about individual polyphenols, for more numerous samples predicted by the experimental design, the application of rapid and low-cost alternative methods was recommended [41]. The content of free and bound polyphenolic compounds ranged from 16.83–36.87 mg GAE/g d.m. and 4.12–9.06 mg GAE/g d.m., respectively (Table 2). Similar free and bound polyphenols value ranges were also noted in the purple potato and dry pea flour extrudates investigated by Nayak et al. [42]. Moreover, the extrusion of different sorghum genotypes also showed higher content of free polyphenols compared to bounded polyphenolic compounds [43]. As anticipated, the control sample recorded lower values of Free PPs and Bound PPs compared to extrudates supplemented with chicory root (8.39 mg GAE/g d.m. of free polyphenols and 2.45 mg GAE/g d.m. of bound polyphenolic compounds) (Table 2). This trend can be justified by the fact that chicory root is a rich source of polyphenols. According to Nwafor et al. [44], the most abundant polyphenols in chicory roots are coumaric, caffeic, chlorogenic, and protocatechuic acids.

The measurement of the antioxidant activity of the biological samples largely depends upon the free radical or the oxidants used in the assays and the degree and type of antioxidants. Hence, it is important to use different antioxidant assays instead of relying on a single assay to assess and compare the antioxidant activity of the extrudates. Synergistic effects and concentration may also change the results that are not observed when the individual constituents are tested.

The antioxidant activity against DPPH• radicals in the fraction of free polyphenolic compounds was in the range of 0.39 mmol TE/g d.m. to 1.03 mmol TE/g d.m., while in the fraction of bound polyphenolic compounds, the activity was recorded in the range of 0.01 mmol TE/g d.m. to 0.06 mmol TE/g d.m. (Table 2). The control sample noted 0.11 mmol TE/g d.m. for DPPHF and 0.00 mmol TE/g d.m. for DPPHB. The highest antioxidant activity was determined in sample 3, containing 35.9% chicory root (1.03 and 0.06 mmol TE/g d.m for DPPHF and DPPHB, respectively).

The antioxidant activity against ABTS+• radicals was in the range of 254.96 mmol TE/g d.m. up to 498.21 mmol TE/g d.m. for the ABTSF, i.e., of 57.37 mmol TE/g d.m. up to 96.47 mmol TE/g d.m for the ABTSB. The control sample recorded 85.73 mmol TE/g d.m. and 35.36 mmol TE/g d.m. for ABTSF and ABTSB, respectively.

The reducing capacity was in ranges of 45.62–108.97 mmol TE/g d.m. for RCF, i.e., 1.87–5.14 mmol TE/g d.m. for RCB (Table 2), while the control showed 14.85 mmol TE/g d.m. for RCF, i.e., 0.76 mmol TE/g d.m. for RCB (Table 2).

The gluten-free chicory-enriched snacks were reported to have higher antioxidant capacity when compared to the control sample. Chicory root addition positively contributed to the increased antioxidative capacity, which is confirmed by the high positive correlations between the chicory root content and antioxidative capacity (R^2^ = 0.89; 0.80; 0.94; 0.82; 0.90, and 0.92 for DPPHF, DPPHB, ABTSF, ABTSB, RCF, and RCB, respectively). The addition of polyphenolic-rich materials also improved the extrudate’s antioxidant capacity in the studies of Vallée et al. [45] and Igual et al. [46], when compared to the control samples.

**Figure 3:**
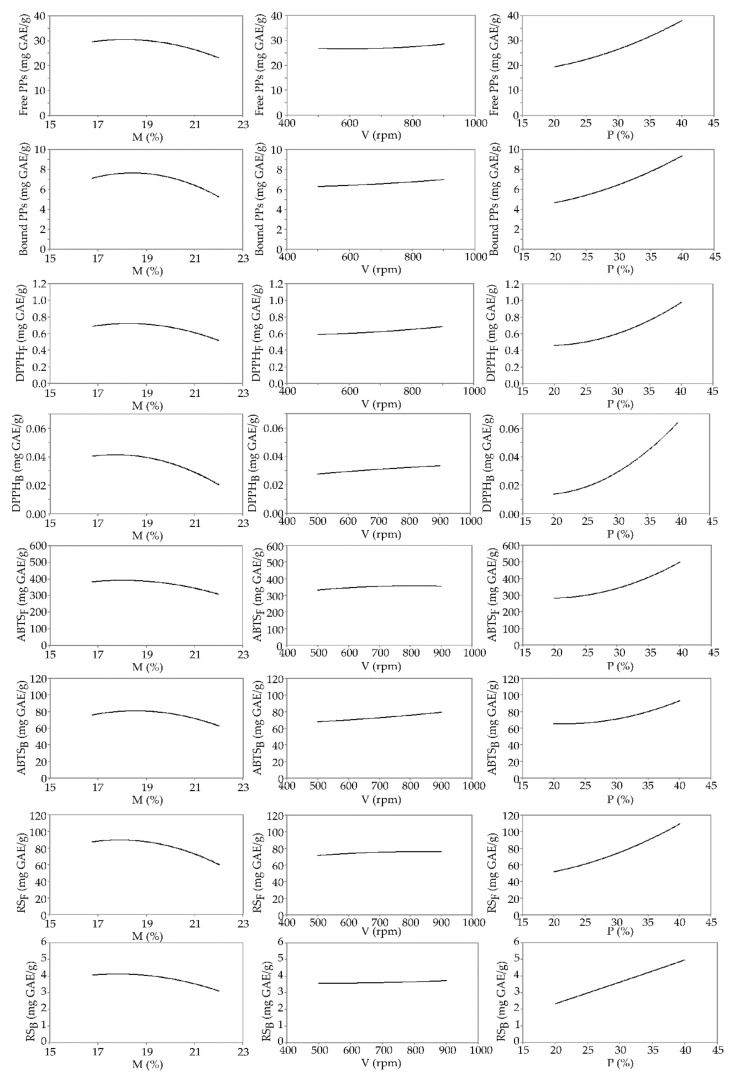
Influence of extrusion conditions (M—moisture content; V—screw speed; P—chicory root content) on polyphenols (PPs) content and antioxidative activity. Index F represent fraction of free polyphenols, and index B represents fraction of bound polyphenols.

The impact of the M, V, and P variables was computed using Yoon’s local sensitivity formula, and the relative influence of these variables on the total phenolic content and antioxidant capacity is shown in Figure 3.

Increased moisture negatively affected the content of polyphenolic compounds (Figure 3). High moisture content possibly favors the decarboxylation of phenolic acids [46]. Moreover, the polymerization of polyphenolic compounds can occur during extrusion, reducing its extractability and, consequently, the antioxidant activity [47]. Similar observations were noted by Yaǧci and Göǧüş [48], where the content of polyphenolic compounds decreased with the increasing moisture content of corn-based extrudates enriched with broccoli flour.

The increase in the screw speed raised the content of PPs (Figure 3), probably due to the release of polyphenolic compounds from the matrixes [49]. Furthermore, high screw speeds (400 rpm) may accelerate the formation of products of non-enzymatic browning of the phenolic structure [50]. Moreover, the increase in screw speed could shorten the time of exposure of polyphenolic compounds to thermal destruction, due to which they remain largely preserved, as noted by Natabirwa et al. [51].

Chicory root supplementation contributed to an increase in polyphenolic compounds (Figure 3) due to the richness of chicory root in polyphenolic compounds [5]. High positive correlations were determined between chicory root and polyphenolic contents (r = 0.92 for Free PPs and r = 0.86 for Bound PPs, *p* < 0.05). The control sample made from pure rice flour was indigent in polyphenolic content due to the loss of these compounds during rice milling [52].

The increase in the screw speed and chicory root content had a positive effect on the antioxidant and reduction capacity of the extruded products (Figure 3). Such extrusion conditions increased the polyphenolic content of the extrudates, which are directly related to the antioxidative activity and reducing capacity, while moisture showed the opposite effect [53].

### 3.4. Mineral Elements

Chicory root might be a relevant ingredient in foods due to the notable presence of minerals. These elements have been considered important traits for a balanced diet [5]. Chicory root proved to be rich in K, Na, Ca, and Mg, while significant amounts of Fe, Zn, Mg, and Cu were also noted (Table 3). These results are in agreement with those reported by Nwafor et al. [44] and Zarroug et al. [54].

The mineral contents in the obtained extrudates ranged within the following limits: 385.94–713.51 mg/kg for Ca, 1835.95–3018.60 mg/kg for K, 427.24–564.56 mg/kg for Mg, 1145.93–2076.30 mg/kg for Na, 24.21–54.13 mg/kg for Fe, 6.07–7.36 mg/kg for Mn, 12.16–15.75 mg/ kg for Zn, and 2.26–3.76 mg/kg for Cu. The achieved values of minerals are close to those corresponding to the recommended daily allowance (RDA) of 1000 mg for Ca, 3500 mg for K, 350 mg for Mg, 2400 mg for Na, 15 mg for Fe, 5 mg for Mn, 15 mg for Zn, and 2 mg for Cu [55].

**Figure 4:**
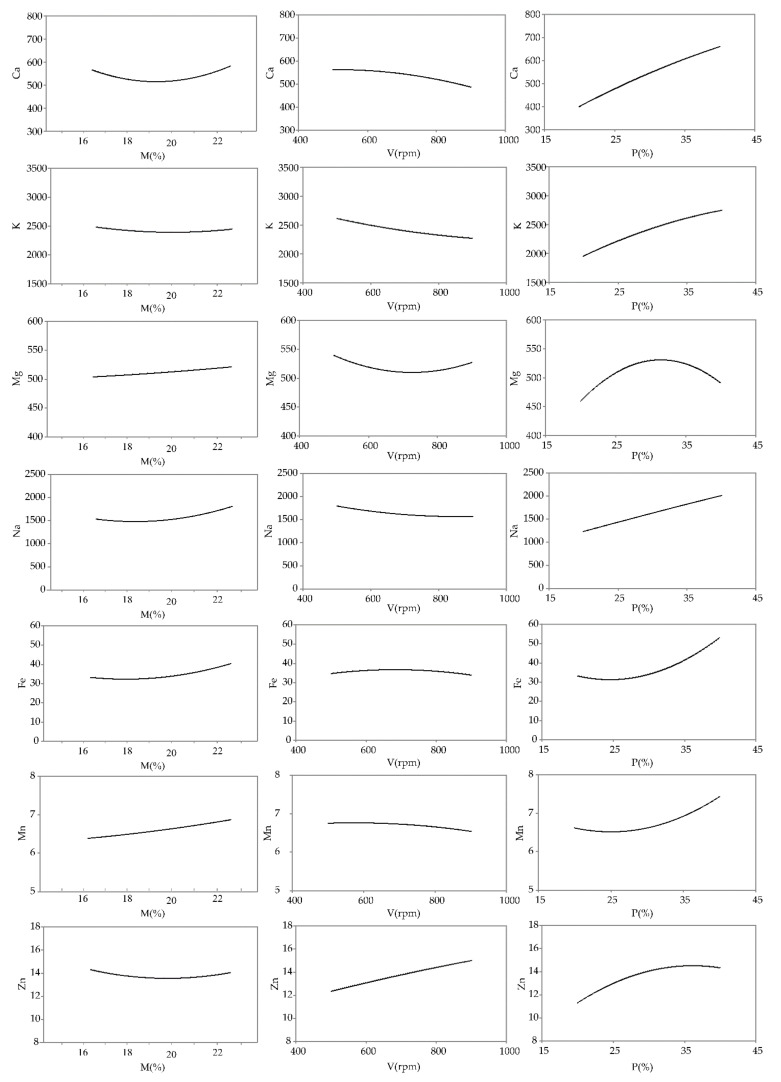
Influence of extrusion conditions (M—moisture content; V—screw speed; P—chicory root content) on mineral content.

The influence of the M, V, and P variables was calculated utilizing Yoon’s local sensitivity formula, and the relative influence of these variables on the mineral content is shown in Figure 4.

Moisture increase had a positive effect on the mineral content (Figure 4). Similar observations were made by Danbaba et al. [56], who investigated the mineral content of rice extrudates supplemented with peas. The increase in mineral content in the extrudates might be due to their accumulation in the water [57].

Increasing the screw speed negatively affected the mineral content, except Zn (Figure 4). Increased screw speed can promote the binding of zinc ions with fibers (such as inulin) more efficiently, which might preserve this element during extrusion [58]. Screw speeds above 700 rpm resulted in increased Mg (Figure 4), which is potentially a consequence of the destruction of anti-nutritive compounds (such as tannins and phytates that build insoluble complexes with minerals), which favorably affects the availability of minerals [59].

Chicory root improved the mineral content of the extrudates when compared to the control sample (Figure 4). This observation might be related to the richness of chicory roots in minerals [5]. Positive correlations were noted between chicory root addition and all of the investigated minerals (r = 0.64 to 0.92, *p* < 0.05). The decreased content of Mg after the addition of more than 30% chicory root may be related to fibers (inulin), which might act as chelating agents, interfering with the extraction of Mg [60].

### 3.5. Artificial Neural Network

The optimal numbers of neurons in the hidden layers were chosen according to ANN performance (network MLP) with the aim of reaching high values of R^2^. The quality of the ANN models was tested through the coefficient of determination (R^2^), which should be close to 1 [61].

The optimal number of neurons in the hidden layer for inulin content prediction was four (MLP 3-4-1 network), according to the highest value of R2 (0.97). The optimal network was constructed using the BFGS 1000 algorithm, while the activation functions in hidden and output layers were exponential and identical functions [23,24,25,26]. A similar R2 value (up to 0.94) of the ANN model was noted in the study of Pandiselvam et al. [62] during the analysis of the extrusion conditions (screw speed, barrel temperature, and formulation) on different product characteristics.

The optimal number of neurons in the hidden layer for antioxidant activity prediction was three (MLP 3-3-8 network), according to the high values of R2 (0.87, 0.81, 0.85, 0.82, 0.91, 0.66, 0.83, and 0.78, for the prediction of Free PPs, Bound PPs, DDPHF, DDPHB, ABTSF, ABTSB, RCF, and RCB, respectively). The optimal network was constructed using the BFGS 57 algorithm, while the activation functions in hidden and output layers were hyperbolic tangents and identical functions [23,24,25,26]. The R2 values obtained within this study follow the results of Pandey et al., who investigated the antioxidant activity and the total phenolic contents of raw banana and defatted soy composite extrudates (R2 = 0.99 and R2 = 0.96, respectively) [63].

The optimal number of neurons in the hidden layer for sesquiterpene lactones prediction was nine (MLP 3-9-4 network), according to the high values of R2 (1.00, 0.99, 0.99, and 0.99, for prediction of lactucin, lactucopyrin, dihydrolactucin, and dihydorolactucopyrin, respectively). The optimal network was constructed using the BFGS 771 algorithm, while the activation functions in hidden and output layers were hyperbolic tangents and identical functions [23,24,25,26]. This study represents one of the first investigations in the ANN prediction of the SLs content in extruded food, while high R2 values indicate the excellent predictive performance of the ANN model.

The optimal number of neurons in the hidden layer for mineral content prediction was nine (MLP 3-9-8 network), according to the high values of R2 (0.99, 0.99, 0.92, 0.99, 0.99, 0.95, 0.99, and 0.99, for the prediction of Ca, K, Mg, Na, Fe, Mn, Zn, and Cu content, respectively). The optimal network was constructed using the BFGS 1000 algorithm, while the activation functions in hidden and output layers were hyperbolic tangents and identical functions [23,24,25,26]. Kothakota et al. noted R2 up to 0.99 during the ANN prediction of mineral content (Ca, P, and Fe) in enzymatic milled rice [64].

## 4. Conclusions

The extrusion process conditions (moisture content, screw speed, and chicory root content) affected the bioactive profile and antioxidant activity of rice-based chicory-enriched snacks.

Increased moisture content contributed to an increase in SLs and minerals, while high screw speed enhanced the polyphenol content. Chicory root addition contributed to the improved bioactive features of the obtained snacks, compared to the control sample in terms of inulin, SLs, and polyphenols, as well as antioxidant activities. Mineral content was also positively influenced by chicory root addition.

The achieved results presented the important impact of extrusion on the bioactive profile of the obtained snacks and promoted chicory root as an attractive food ingredient in terms of functionality. Furthermore, the consumers’ acceptability of novel gluten-free chicory snacks will be the goal of future investigation.

## Figures and Tables

**Table 1:** Inulin and sesquiterpene lactones (SLs) contents in extrudates and control sample (CS).

CCD Design								
					SLs			
S.No.	M, %	V, rpm	P, %	Inulin, %	Lactucin, µg/g	Lactucopicrin, µg/g	Dihydrolactucin, µg/g	Dihydrolactucopicrin, µg/g
1	21.2	820	35.9	5.52 ± 0.26 ^c^	7.35 ± 0.89 ^e^	4.15 ± 0.49 ^gh^	4.15 ± 0.49 ^gh^	9.79 ± 0.97 ^h^
2	19.4	700	30.0	7.26 ± 0.16 ^e^	4.29 ± 0.56 ^cd^	2.74 ± 0.34 ^ef^	2.64 ± 0.34 ^de^	5.09 ± 0.57 ^de^
3	17.6	820	35.9	7.15 ± 0.06 ^e^	9.13 ± 0.93 ^f^	4.61 ± 0.41 ^h^	4.61 ± 0.41 ^h^	9.47 ± 0.86 ^h^
4	19.4	700	30.0	7.55 ± 0.41 ^e^	4.35 ± 0.47 ^cd^	2.72 ± 0.29 ^ef^	2.62 ± 0.29 ^de^	4.97 ± 0.43 ^de^
5	21.2	820	24.1	4.28 ± 0.42 ^b^	1.92 ± 0.21 ^b^	1.13 ± 0.13 ^ab^	1.13 ± 0.13 ^ab^	2.45 ± 0.25 ^b^
6	22.5	700	30.0	9.17 ± 0.18 ^g^	4.97 ± 0.52 ^d^	2.89 ± 0.12 ^ef^	2.89 ± 0.12 ^ef^	5.68 ± 0.46 ^ef^
7	19.4	900	30.0	8.39 ± 0.14 ^f^	4.77 ± 0.61 ^d^	2.53 ± 0.23 ^de^	2.53 ± 0.23 ^de^	4.76 ± 0.51 ^cde^
8	17.6	820	24.1	5.64 ± 1.05 ^c^	3.97 ± 0.38 ^cd^	1.64 ± 0.16 ^bc^	1.64 ± 0.16 ^bc^	3.68 ± 0.33 ^bcd^
9	19.4	700	30.0	7.77 ± 0.55 ^e^	4.53 ± 0.50 ^cd^	2.50 ± 0.36 ^de^	2.50 ± 0.36 ^de^	5.05 ± 0.48 ^de^
10	21.2	580	24.1	4.18 ± 0.49 ^b^	3.12 ± 0.43 ^bc^	1.94 ± 0.20 ^cd^	1.94 ± 0.20 ^cd^	4.16 ± 0.53 ^cde^
11	16.3	700	30.0	5.36 ± 0.10 ^c^	5.05 ± 0.57 ^d^	2.22 ± 0.41 ^cde^	2.22 ± 0.41 ^cde^	4.54 ± 0.40 ^cde^
12	19.4	700	40.0	9.49 ± 0.69 ^g^	7.28 ± 0.75 ^e^	4.18 ± 0.52 ^h^	4.18 ± 0.52 ^h^	8.61 ± 0.76 ^gh^
13	19.4	500	30.0	7.61 ± 0.64 ^e^	0.51 ± 0.12 ^a^	0.56 ± 0.10 ^a^	0.56 ± 0.10 ^a^	0.49 ± 0.09 ^a^
14	19.4	700	30.0	7.66 ± 0.66 ^e^	4.42 ± 0.34 ^cd^	2.62 ± 0.44 ^de^	2.62 ± 0.44 ^de^	5.14 ± 0.55 ^de^
15	17.6	580	24.1	6.58 ± 0.14 ^d^	2.16 ± 0.23 ^b^	1.52 ± 0.17 ^bc^	1.52 ± 0.17 ^bc^	3.28 ± 0.39 ^bc^
16	21.2	580	35.9	4.23 ± 0.03 ^b^	8.72 ± 0.88 ^f^	5.52 ± 0.64 ^i^	5.52 ± 0.64 ^i^	12.39 ± 0.99 ^i^
17	17.6	580	35.9	10.10 ± 0.27 ^h^	4.64 ± 0.63 ^d^	3.38 ± 0.39 ^fg^	3.38 ± 0.39 ^fg^	5.38 ± 0.67 ^e^
18	19.4	700	20.0	3.29 ± 0.02 ^a^	6.40 ± 0.72 ^e^	2.86 ± 0.38 ^ef^	2.86 ± 0.38 ^ef^	7.21 ± 0.78 ^fg^
19	19.4	700	30.0	7.42 ± 0.64 ^e^	4.46 ± 0.54 ^cd^	2.63 ± 0.40 ^de^	2.63 ± 0.40 ^de^	5.22 ± 0.72 ^de^
20	19.4	700	30.0	7.59 ± 1.31 ^e^	4.45 ± 0.47 ^cd^	2.60 ± 0.48 ^de^	2.60 ± 0.48 ^de^	4.97 ± 0.68 ^de^
CS	18.0	800	00.0	n.d.	n.d.	n.d.	n.d.	n.d.
CV				2.87	1.93	3.31	1.98	1.93

CCD—central composite design; S.no.—Sample number; M—moisture content; SS—screw speed; P—chicory root flour content; SLs—sesquiterpene lactones; CS—control sample; n.d.—not detected; CV—coefficient of variation for six central points (samples 2, 4, 9, 14, 19, and 20). Values in the same column marked with different letters were statistically significantly (*p* < 0.05) different (Tukey HSD test).

**Table 2:** Contents of free (Free PPs) and bound (Bound PPs) (poly) phenolics (PPs), and the antioxidant capacity tests for both free and bound (polyphenolics) by the DPPH and ABTS methods. Total reducing capacity (RC) test for free (RCF) and bound phenolics (RCB) for extrudates and the control (CS).

CCD Design											
S.No.	M, %	V, rpm	P, %	Free PPs, mg GAE/g d.w.	Bound PPs, mg GAE/g d.w.	DDPHF, mmol TE/g d.w.	DDPHB, mmol TE/g d.w.	ABTSF, mmol TE/g d.w.	ABTSB, mmol TE/g d.w.	RCF, mmol TE/g d.w.	RCB, mmol TE/g d.w.
1	21.2	820	35.9	33.26 ± 0.64 ^h^	8.13 ± 0.06 ^fg^	0.79 ± 0.00 ^efg^	0.05 ± 0.00 ^f^	400.17 ± 28.52 ^efg^	80.41 ± 3.26 ^bc^	91.99 ± 7.06 ^efg^	4.54 ± 0.00 ^h^
2	19.4	700	30.0	27.12 ± 0.13 ^efg^	6.96 ± 0.11 ^def^	0.61 ± 0.07 ^cde^	0.03 ± 0.00 ^d^	356.28 ± 12.64 ^cdef^	71.28 ± 3.69 ^bc^	79.54 ± 4.16 ^cdefg^	3.97 ± 0.00 ^f^
3	17.6	820	35.9	36.87 ± 0.98 ^i^	9.06 ± 0.32 ^g^	1.03 ± 0.00 ^h^	0.06 ± 0.00 ^g^	473.69 ± 12.97 ^gh^	96.47 ± 15.02 ^c^	108.97 ± 2.71 ^g^	5.14 ± 0.01 ^j^
4	19.4	700	30.0	27.01 ± 0.02 ^efg^	6.57 ± 0.24 ^def^	0.62 ± 0.07 ^cde^	0.03 ± 0.00 ^d^	347.23 ± 11.54 ^cdef^	70.79 ± 1.29 ^bc^	79.1 ± 3.15 ^cdefg^	3.76 ± 0.00 ^f^
5	21.2	820	24.1	23.11 ± 0.54 ^cd^	5.51 ± 0.12 ^bcd^	0.54 ± 0.09 ^bcd^	0.02 ± 0.00 ^c^	313.69 ± 2.69 ^bcde^	65.97 ± 13.06 ^bc^	60.12 ± 7.56 ^bcdef^	3.11 ± 0.00 ^d^
6	22.5	700	30.0	20.47 ± 0.06 ^c^	4.25 ± 0.57 ^bc^	0.48 ± 0.01 ^bc^	0.01 ± 0.00 ^b^	289.36 ± 24.56 b^cd^	61.17 ± 4.15 ^bc^	51.89 ± 2.61 ^bc^	2.83 ± 0.00 ^c^
7	19.4	900	30.0	24.55 ± 0.69 ^de^	6.05 ± 0.09 ^de^	0.55 ± 0.01 ^bcd^	0.02 ± 0.00 ^c^	316.16 ± 5.25 ^bcde^	67.69 ± 5.50 ^bc^	62.06 ± 2.97 ^bcde^	3.09 ± 0.00 ^d^
8	17.6	820	24.1	26.98 ± 0.39 ^efg^	6.90 ± 0.25 ^def^	0.60 ± 0.00 ^de^	0.03 ± 0.00 ^d^	340.17 ± 23.69 ^bcde^	89.33 ± 16.58 ^bc^	77.14 ± 9.88 ^bcdefg^	3.47 ± 0.01 ^e^
9	19.4	700	30.0	26.68 ± 0.21 ^efg^	6.85 ± 0.04 ^def^	0.63 ± 0.00 ^cde^	0.03 ± 0.00 ^d^	353.69 ± 16.87 ^cdef^	69.79 ± 2.58 ^bc^	77.47 ± 1.65 ^cdefg^	3.86 ± 0.02 ^f^
10	21.2	580	24.1	20.81 ± 0.03 ^c^	4.41 ± 0.39 ^bc^	0.48 ± 0.07 ^bc^	0.01 ± 0.00 ^b^	279.36 ± 21.49 ^bc^	62.09 ± 7.28 ^bc^	53.71 ± 8.63 ^bcd^	2.91 ± 0.00 ^c^
11	16.3	700	30.0	28.54 ± 0.03 ^g^	7.01 ± 0.03 ^def^	0.66 ± 0.02 ^cde^	0.04 ± 0.00 ^e^	374.63 ± 13.64 ^def^	75.87 ± 2.14 ^bc^	84.65 ± 9.23 ^defg^	3.94 ± 0.00 ^f^
12	19.4	700	40.0	36.14 ± 0.87 ^i^	8.94 ± 0.01 ^g^	0.91 ± 0.01 ^gh^	0.06 ± 0.00 ^g^	498.21 ± 14.36 ^h^	91.63 ± 2.41 ^bc^	103.89 ± 1.47 ^g^	4.69 ± 0.02 ^i^
13	19.4	500	30.0	25.77 ± 0.36 ^def^	6.13 ± 0.68 ^de^	0.56 ± 0.00 ^bcd^	0.03 ± 0.00 ^d^	324.61 ± 2.57 ^bcde^	67.48 ± 2.11 ^bc^	71.62 ± 9.46 ^bcdef^	3.51 ± 0.00 ^e^
14	19.4	700	30.0	27.36 ± 1.06 ^efg^	7.16 ± 0.01 ^def^	0.61 ± 0.00 ^cde^	0.03 ± 0.00 ^d^	351.98 ± 2.65 ^cdef^	72.24 ± 5.79 ^bc^	78.73 ± 2.65 ^cdefg^	3.92 ± 0.00 ^f^
15	17.6	580	24.1	23.55 ± 0.28 ^d^	5.81 ± 0.08 ^cde^	0.53 ± 0.03 ^bcd^	0.02 ± 0.00 ^c^	301.21 ± 14.65 ^bcd^	65.26 ± 1.36 ^bc^	62.35 ± 7.45 ^bcde^	3.15 ± 0.00 ^d^
16	21.2	580	35.9	32.05 ± 0.43 ^h^	8.01 ± 0.09 ^fg^	0.71 ± 0.03 ^def^	0.05 ± 0.00 ^f^	395.27 ± 14.57 ^efg^	78.25 ± 11.69 ^bc^	91.43 ± 6.93 ^efg^	4.15 ± 0.03 ^g^
17	17.6	580	35.9	34.56 ± 0.91 ^hi^	8.32 ± 0.11 ^fg^	0.84 ± 0.01 ^fgh^	0.04 ± 0.00 ^e^	436.11 ± 23.14 ^fgh^	81.24 ± 6.54 ^bc^	96.36 ± 5.87 ^fg^	4.69 ± 0.00 ^i^
18	19.4	700	20.0	16.83 ± 0.07 ^b^	4.12 ± 0.08 ^b^	0.39 ± 0.00 ^b^	0.01 ± 0.00 ^b^	254.96 ± 6.4^3 b^	57.37 ± 0.12 ^b^	45.62 ± 2.32 ^b^	1.87 ± 0.03 ^b^
19	19.4	700	30.0	26.96 ± 0.13 ^efg^	6.87 ± 0.21 ^def^	0.61 ± 0.06 ^cde^	0.03 ± 0.00 ^d^	352.21 ± 17.61 ^cdef^	70.06 ± 5.69 ^bc^	78.14 ± 0.39 ^cdefg^	3.69 ± 0.00 ^f^
20	19.4	700	30.0	27.08 ± 0.09 ^efg^	6.99 ± 0.56 ^def^	0.62 ± 0.00 ^cde^	0.03 ± 0.00 ^d^	353.28 ± 29.64 ^cdef^	70.31 ± 6.91 ^bc^	80.11 ± 5.28 ^cdefg^	3.76 ± 0.04 ^f^
CS	18.0	800	00.0	8.39 ± 0.11 ^a^	2.45 ± 0.35 ^a^	0.11 ± 0.01 ^a^	0.00 ± 0.00 ^a^	85.73 ± 0.97 ^a^	35.36 ± 1.05 ^a^	14.85 ± 0.41 ^a^	0.76 ± 0.01 ^a^
CV				0.82	2.84	1.68	0.00	0.85	1.28	1.21	2.81

CCD—central composite design; S.no.—Sample number; M—moisture content; V—screw speed; P—chicory root flour content; PPs—polyphenols; DDPHF—DPPH antioxidant activity of free polyphenolic fraction; DDPHB_—_DPPH antioxidant activity of bound polyphenolic fraction; ABTSF—ABTS antioxidant activity of free polyphenolic fraction; ABTSB—ABTS antioxidant activity of bound polyphenolic fraction; RCF_—_reducing capacity of free polyphenolic fraction; RCB—reducing capacity of bound polyphenolic fraction; GAE—gallic acid equivalent; TE—trolox equivalent; d.w.—dry weight; CS—control sample; CV— coefficient of variation for six central points (samples 2, 4, 9, 14, 19, and 20). Values in the same column marked with different letters were statistically significantly (*p* < 0.05) different (Tukey HSD test).

**Table 3:** Mineral contents in extrudates and control sample (CS).

CCD Design											
S.No.	M, %	V, rpm	P, %	Ca	K	Mg	Na	Fe	Mn	Zn	Cu
1	21.2	820	35.9	639.29 ± 1.32 ^j^	2242.72 ± 4.32 ^f^	501.48 ± 0.98 ^de^	2076.30 ± 3.03 ^j^	54.13 ± 0.26 ^i^	7.36 ± 0.06 ^de^	15.75 ± 0.22 ^i^	3.75 ± 0.09 ^f^
2	19.4	700	30.0	534.07 ± 1.04 ^g^	2292.48 ± 3.13 ^h^	512.78 ± 0.83 ^fg^	1562.49 ± 2.76 ^e^	33.63 ± 0.13 ^e^	6.91 ± 0.11 ^bcde^	14.77 ± 0.37 ^ghi^	2.83 ± 0.03 ^bcde^
3	17.6	820	35.9	427.90 ± 1.12 ^d^	2159.61 ± 2.59 ^d^	514.25 ± 1.01 ^fg^	1358.71 ± 2.02 ^d^	28.41 ± 0.11 ^c^	6.40 ± 0.09 ^bc^	15.59 ± 0.45 ^i^	2.27 ± 0.11 ^bc^
4	19.4	700	30.0	535.51 ± 2.01 ^g^	2290.68 ± 3.24 ^h^	539.55 ± 0.76 ^fg^	1561.92 ± 2.14 ^e^	35.25 ± 0.27 ^e^	6.93 ± 0.09 ^bcde^	14.76 ± 0.51 ^ghi^	2.95 ± 0.09 ^bcde^
5	21.2	820	24.1	483.27 ± 1.00 ^f^	2214.95 ± 2.11 ^e^	534.14 ± 1.12 ^ij^	1398.47 ± 1.98 ^d^	31.04 ± 0.31 ^d^	6.90 ± 0.07 ^bcde^	11.21 ± 0.20 ^bc^	2.26 ± 0.21 ^bc^
6	22.5	700	30.0	556.71 ± 0.98 ^h^	2544.48 ± 2.27 ^j^	545.94 ± 1.22 ^k^	1700.58 ± 2.04 ^fg^	34.47 ± 0.44 ^e^	6.53 ± 0.10 ^bcd^	15.60 ± 0.11 ^i^	2.78 ± 0.17 ^bcde^
7	19.4	900	30.0	533.90 ± 1.03 ^g^	2565.08 ± 3.01 ^k^	533.04 ± 0.99 ^jk^	1702.68 ± 2.15 ^fg^	35.18 ± 0.34 ^e^	6.84 ± 0.12 ^bcde^	15.48 ± 0.27 ^hi^	3.15 ± 0.23 ^def^
8	17.6	820	24.1	417.43 ± 1.15 ^c^	2070.44 ± 1.09 ^c^	487.20 ± 0.73 ^c^	1242.16 ± 1.72 ^c^	26.05 ± 0.25 ^bc^	6.07 ± 0.07 ^b^	14.50 ± 0.35 ^gh^	2.29 ± 0.31 ^b^
9	19.4	700	30.0	531.03 ± 1.02 ^g^	2293.79 ± 1.56 ^h^	508.63 ± 1.01 ^fg^	1560.18 ± 1.84 ^e^	36.34 ± 0.61 ^e^	6.91 ± 0.09 ^bcde^	15.01 ± 0.19 ^ghi^	2.88 ± 0.14 ^bcde^
10	21.2	580	24.1	482.28 ± 0.89 ^f^	2240.59 ± 1.48 ^f^	510.00 ± 0.94 ^ef^	1729.49 ± 2.01 ^g^	26.25 ± 0.23 ^bc^	6.43 ± 0.10 ^bc^	10.72 ± 0.29 ^b^	3.07 ± 0.19 ^def^
11	16.3	700	30.0	563.83 ± 1.17 ^i^	2526.92 ± 3.22 ^i^	534.87 ± 1.04 ^ij^	1693.07 ± 1.87 ^fg^	36.02 ± 0.56 ^e^	6.44 ± 0.11 ^bc^	11.32 ± 0.35 ^bc^	2.63 ± 0.09 ^bcd^
12	19.4	700	40.0	713.51 ± 2.09 ^k^	3018.60 ± 4.02 ^n^	526.52 ± 0.73 ^hi^	2074.80 ± 2.90 ^j^	51.11 ± 0.72 ^h^	7.32 ± 0.07 ^e^	13.30 ± 0.27 ^ef^	3.75 ± 0.07 ^f^
13	19.4	500	30.0	555.55 ± 1.23 ^h^	2525.85 ± 3.12 ^i^	564.65 ± 1.12 ^l^	1668.48 ± 1.78 ^f^	36.62 ± 0.45 ^e^	6.59 ± 0.12 ^bcde^	12.48 ± 0.41 ^de^	2.96 ± 0.08 ^cdef^
14	19.4	700	30.0	532.15 ± 1.11 ^g^	2285.53 ± 2.12 ^h^	552.90 ± 1.01 ^fg^	1560.16 ± 1.09 ^e^	36.77 ± 0.37 ^e^	6.90 ± 0.15 ^bcde^	14.91 ± 0.61 ^ghi^	2.86 ± 0.11 ^bcde^
15	17.6	580	24.1	472.51 ± 0.99 ^e^	2260.06 ± 1.87 ^g^	522.93 ± 0.95 ^gh^	1381.76 ± 1.23 ^d^	24.21 ± 0.22 ^b^	6.87 ± 0.07 ^bcde^	12.16 ± 0.37 ^cd^	2.43 ± 0.12 ^bcd^
16	21.2	580	35.9	644.01 ± 2.01 ^j^	2777.22 ± 2.35 ^l^	493.60 ± 0.84 ^cd^	1899.85 ± 2.34 ^h^	46.03 ± 0.48 ^g^	7.09 ± 0.09 ^cde^	14.12 ± 0.59 ^fg^	3.76 ± 0.21 ^f^
17	17.6	580	35.9	643.34 ± 1.92 ^j^	2824.91 ± 2.47 ^m^	507.18 ± 1.01 ^ef^	2027.11 ± 2.76 ^i^	43.99 ± 0.58 ^g^	7.29 ± 0.11 ^de^	14.20 ± 0.63 ^fg^	3.49 ± 0.31 ^ef^
18	19.4	700	20.0	385.94 ± 1.35 ^b^	1835.95 ± 1.03 ^b^	427.24 ± 0.78 ^b^	1145.93 ± 1.03 ^b^	41.78 ± 0.33 ^f^	6.81 ± 0.08 ^bcde^	12.60 ± 0.43 ^de^	3.08 ± 0.19 ^def^
19	19.4	700	30.0	532.69 ± 1.22 ^g^	2295.36 ± 2.25 ^h^	506.46 ± 1.17 ^fg^	1560.14 ± 1.54 ^e^	35.45 ± 0.28 ^e^	6.92 ± 0.07 ^bcde^	14.68 ± 0.52 ^ghi^	2.82 ± 0.11 ^bcde^
20	19.4	700	30.0	533.35 ± 1.24 ^g^	2293.32 ± 2.11 ^h^	528.88 ± 0.72 ^fg^	1562.28 ± 1.86 ^e^	35.14 ± 0.31 ^e^	6.93 ± 0.08 ^bcde^	14.47 ± 0.47 ^ghi^	2.98 ± 0.10 ^bcde^
CS	18.0	800	00.0	43.82 ± 0.26 ^a^	934.98 ± 0.89 ^a^	380.18 ± 0.45 ^a^	56.10 ± 0.37 ^a^	3.42 ± 0.41 ^a^	4.79 ± 0.07 ^a^	9.91 ± 0.06 ^a^	1.08 ± 0.05 ^a^
CV				0.29	0.15	3.58	0.07	3.09	0.18	1.27	2.25

CCD—central composite design; S.no.—Sample number; M—moisture content; SS—screw speed; P—chicory root flour content; CS—control sample; CV—coefficient of variation for six central points (samples 2, 4, 9, 14, 19, and 20). Values in the same column marked with different letters were statistically significantly (*p* < 0.05) different (Tukey HSD test).

## Data Availability

Authors can confirm that all relevant data are included in the article and/or its Supplementary Material files. The datasets generated during and/or analyzed during the current study are available from the corresponding author upon reasonable request.

## References

[1] Výrostková J., Regecová I., Zigo F., Marcinčák S., Kožárová I., Kováčová M., Bertová D. (2022). Detection of Gluten in Gluten-Free Foods of Plant Origin. Foods.

[2] Taylor J.R.N., Taylor J., Campanella O.H., Hamaker B.R. (2016). Functionality of the storage proteins in gluten-free cereals and pseudocereals in dough systems. J. Cereal Sci..

[3] Torbica A., Hadnadev M., Dapčević T. (2010). Rheological, textural and sensory properties of gluten-free bread formulations based on rice and buckwheat flour. Food Hydrocoll..

[4] Arribas C., Cabellos B., Cuadrado C., Guillamón E., Pedrosa M.M. (2019). Bioactive compounds, antioxidant activity, and sensory analysis of rice-based extruded snacks-like fortified with bean and carob fruit flours. Foods.

[5] Perović J., Tumbas Šaponjac V., Kojić J., Krulj J., Moreno D.A., García-Viguera C., Bodroža-Solarov M., Ilić N. (2021). Chicory (*Cichorium intybus* L.) as a food ingredient—Nutritional composition, bioactivity, safety, and health claims: A review. Food Chem..

[6] Pouille C.L., Jegou D., Dugardin C., Cudennec B., Ravallec R., Hance P., Rambaud C., Hilbert J.L., Lucau-Danila A. (2020). Chicory root flour—A functional food with potential multiple health benefits evaluated in a mice model. J. Funct. Foods.

[7] Nishimura M., Ohkawara T., Kanayama T., Kitagawa K., Nishimura H., Nishihira J. (2015). Effects of the extract from roasted chicory (*Cichorium intybus* L.) root containing inulin-type fructans on blood glucose, lipid metabolism, and fecal properties. J. Tradit. Complement. Med..

[8] Ripoll C., Schmidt M.B., Ilic N., Poulev A., Dey M., Kurmukov G.A., Raskin I. (2007). Anty-inflammatory effects of a sesquiterpene lactone extract from chicory (*Cichorium intybus* L) roots. Nat. Prod. Commun..

[9] Mihaylova D., Vrancheva R., Petkova N., Ognyanov M., Desseva I., Ivanov I., Popova M., Popova A. (2018). Carotenoids, tocopherols, organic acids, charbohydrate and mineral content in different medicinal plant extracts. Z. Naturforsch.-C J. Biosci..

[10] Harrington K.C., Thatcher A., Kemp P.D. (2006). Mineral composition and nutritive value of some common pasture weeds. N. Z. Plant Prot..

[11] Patil S.S., Kaur C. (2018). Current trends in extrusion: Development of functional foods and novel ingredients. Food Sci. Technol. Res..

[12] Morales P., Berrios J.D.J., Varela A., Burbano C., Cuadrado C., Muzquiz M., Pedrosa M.M. (2015). Novel fiber-rich lentil flours as snack-type functional foods: An extrusion cooking effect on bioactive compounds. Food Funct..

[13] Dilrukshi H.N.N., Torrico D.D., Brennan M.A., Brennan C.S. (2022). Effects of extrusion processing on the bioactive constituents, in vitro digestibility, amino acid composition, and antioxidant potential of novel gluten-free extruded snacks fortified with cowpea and whey protein concentrate. Food Chem..

[14] Bokić J., Kojić J., Krulj J., Pezo L., Banjac V., Škrobot D., Tumbas Šaponjac V., Vidosavljević S., Stojkov V., Ilić N. (2022). Development of a Novel Rice-Based Snack Enriched with Chicory Root: Physicochemical and Sensory Properties. Foods.

[15] Perović J., Kojić J., Krulj J., Pezo L., Tumbas Šaponjac V., Ilić N., Bodroža-Solarov M. (2022). Inulin determination by an improved HPLC-ELSD method. Food Anal. Methods.

[16] Schmidt B.M., Ilic N., Poulev A., Raskin I. (2007). Toxicological evaluation of a chicory root extract. Food Chem. Toxicol..

[17] (2008). Animal feeding stuffs—Determination of the Contents of Calcium, Copper, Iron, Magnesium, Manganese, Potassium, Sodium and Zinc—Method Using Atomic Absorption Spectrometry.

[18] Singleton L.V., Rossi A.J. (1965). Colorimetry of total phenolics with phospho¬molybdic-phosphotungstic acid reagents. Am. J. Enol. Vitic..

[19] Gaonkar A.G., Vasisht N., Khare A.R., Sobel R. (2014). Microencapsulation in the Food Industry: A Practical Implementation Guide.

[20] Gironés-Vilaplana A., Mena P., Moreno D.A., García-Viguera C. (2014). Evaluation of sensorial, phytochemical and biological properties of new isotonic beverages enriched with lemon and berries during shelf life. J. Sci. Food Agric..

[21] Mena P., Garcia-Viguera C., Navarro-Rico J., Moreno A.D., Bartual J., Saura D., Marti N. (2011). Phytochemical characterisation for industrial use of pomegranate (*Punica granatum* L.) cultivars grown in Spain. J. Sci. Food Agric..

[22] Oyaizu M. (1986). Studies on products of browning reactions: Antioxidative activities of product of browning reaction prepared from glucosamine. Jpn. J. Nutr..

[23] Kollo T., von Rosen D. (2005). Advanced Multivariate Statistics with Matrices.

[24] Trelea I.C., Raoult-Wack A.L., Trystram G. (1997). Application of neural network modelling for the control of dewatering and impregnation soaking process (osmotic dehydration). Food Sci. Technol. Int..

[25] Mohieddin J., Yinyin W., Ali A., Jing T. (2020). Unsupervised Learning and Multipartite Network Models: A Promising Approach for Understanding Traditional Medicine. Front. Pharmacol..

[26] Basheer I.A., Hajmeer M. (2000). Artificial neural networks: Fundamentals, computing, design, and application. J. Microbiol. Methods.

[27] Yoon Y., Swales G., Margavio T.M. (2017). A Comparison of Discriminant Analysis versus Artificial Neural Networks. J. Oper. Res. Soc..

[28] Coussement P.A.A. (1999). Inulin and oligofructose: Safe intakes and legal status. J. Nutr..

[29] Radovanovic A., Stojceska V., Plunkett A., Jankovic S., Milovanovic D., Cupara S. (2015). The use of dry Jerusalem artichoke as a functional nutrient in developing extruded food with low glycaemic index. Food Chem..

[30] Chaito C., Judprasong K., Puwastien P. (2016). Inulin content of fortified food products in Thailand. Food Chem..

[31] Khuenpet K., Jittanit W., Sirisansaneeyakul S., Srichamnong W. (2017). Inulin powder production from Jerusalem artichoke (Helianthus tuberosus L.) tuber powder and its application to commercial food products. J. Food Process. Preserv..

[32] Ferreira S.M., Capriles V.D., Conti-Silva A.C. (2020). Breakfast cereals with inulin obtained through thermoplastic extrusion: Chemical characteristics and physical and technological properties. LWT.

[33] Sharma P., Gujral H.S. (2013). Extrusion of hulled barley affecting β-glucan and properties of extrudates. Food Bioproc. Technol..

[34] Katsavou I.D., Tsokolar-Tsikopoulos K.C., Eleni P.N., Krokida M.K. (2019). Sensorial, functional, optical and thermal properties of inulin enriched expanded products. Int. Food Res. J..

[35] Tsokolar-Tsikopoulos K.C., Katsavou I.D., Krokida M.K. (2015). The effect of inulin addition on structural and textural properties of extruded products under several extrusion conditions. J. Food Sci. Technol..

[36] Singh R.S., Singh T., Larroche C. (2019). Biotechnological applications of inulin-rich feedstocks. Bioresour. Technol..

[37] Weng H., He L., Zheng J., Li Q., Liu X., Wang D. (2020). Low oral bioavailability and partial gut microbiotic and phase ii metabolism of brussels/witloof chicory sesquiterpene lactones in healthy humans. Nutrients.

[38] Kulkarni C., Kelly A.L., Gough T., Jadhav V., Singh K.K., Paradkar A. (2017). Application of hot melt extrusion for improving bioavailability of artemisinin a thermolabile drug. Drug Dev. Ind. Pharm..

[39] Aberham A., Cicek S.S., Schneider P., Stuppner H. (2010). Analysis of sesquiterpene lactones, lignans, and flavonoids in wormwood (*Artemisia absinthium* L.) using high-performance liquid chromatography (HPLC)-mass spectrometry, reversed phase HPLC, and HPLC-Solid phase extraction-nuclear magnetic resonance. J. Agric. Food Chem..

[40] Willeman H., Hance P., Fertin A., Voedts N., Duhal N., Goossens J., Hilbert J. (2014). A method for the simultaneous determination of chlorogenic acid and sesquiterpene lactone content in industrial chicory root foodstuffs. Sci. World J..

[41] Jandrić Z., Cannavan A. (2017). An investigative study on differentiation of citrus fruit/fruit juices by UPLC-QToF MS and chemometrics. Food Control.

[42] Nayak B., Liu R.H., Berrios J.D.J., Tang J., Derito C. (2011). Bioactivity of Antioxidants in Extruded Products Prepared from Purple Potato and Dry Pea Flours. J. Agric. Food Chem..

[43] dos Santos D’Almeida C.T., Mameri H., dos Santos Menezes N., de Carvalho C.W.P., Queiroz V.A.V., Cameron L.C., Morel M.H., Takeiti C.Y., Ferreira M.S.L. (2021). Effect of extrusion and turmeric addition on phenolic compounds and kafirin properties in tannin and tannin-free sorghum. Food Res. Int..

[44] Nwafor I.C., Shale K., Achilonu M.C. (2017). Chemical composition and nutritive benefits of chicory (*Cichorium intybus*) as an ideal complementary and / or alternative livestock feed supplement. Sci. World J..

[45] Vallée M., Lu X., Narciso J.O., Li W., Qin Y., Brennan M.A., Brennan C.S. (2017). Physical, predictive glycaemic response and antioxidative properties of black ear mashroom (*Auricularia auricula*) extrudates. Plant Foods Hum. Nutr..

[46] Igual M., Chiş M.S., Socaci S.A., Vodnar D.C., Ranga F., Martínez-Monzó J., García-Segovia P. (2021). Effect of Medicago sativa Addition on Physicochemical, Nutritional and Functional Characteristics of Corn Extrudates. Foods.

[47] Brennan C., Brennan M., Derbyshire E., Tiwari B.K. (2011). Effects of extrusion on the polyphenols, vitamins and antioxidant activity of foods. Trends Food Sci. Technol..

[48] Yaǧci S., Göǧüş F. (2008). Response surface methodology for evaluation of physical and functional properties of extruded snack foods developed from food-by-products. J. Food Eng..

[49] Leonard W., Zhang P., Ying D., Fang Z. (2020). Application of extrusion technology in plant food processing byproducts: An overview. Compr. Rev. Food Sci. Food Saf..

[50] Chalermchaiwat P., Jangchud K., Jangchud A., Charunuch C., Prinyawiwatkul W. (2015). Antioxidant activity, free gamma-aminobutyric acid content, selected physical properties and consumer acceptance of germinated brown rice extrudates as affected by extrusion process. LWT-Food Sci. Technol..

[51] Natabirwa H., Nakimbugwe D., Lungáho M., Muyonga J.H. (2018). Optimization of Roba1 extrusion conditions and bean extrudate properties using response surface methodology and multi-response desirability function. LWT.

[52] Boue S.M., Daigle K., Beaulieu J.C., Heiman M. (2019). Rice flour and bran enriched with blueberry polyphenols increases storage stability and decreases arsenic content in bran. Foods.

[53] Olszowy M. (2019). What is responsible for antioxidant properties of polyphenolic compounds from plants?. Plant Physiol. Biochem..

[54] Zarroug Y., Abdelkarim A., Dorra S.T., Hamdaoui G., Felah M.E.L., Hassouna M. (2016). Biochemical characterization of Tunisian *Cichorium Intybus* L. roots and optimization of ultrasonic inulin extraction. Mediter. J. Chem..

[55] Lenntech B.V. (2013). Recommended Daily Intake of Vitamins and Minerals.

[56] Danbaba N., Nkama I., Badau M.H. (2015). Application of response surface methodology (RSM) and central composite design (CCD) to optimize minerals composition of rice-cowpea composite blends during extrusion cooking. Int. J. Food Sci. Nut. Eng..

[57] Camire M.E., Camire A., Krumhar K. (1990). Chemical and nutritional changes in foods during extrusion. Crit. Rev. Food Sci. Nutr..

[58] Bergman C.J., Gualberto D.G., Weber C.W. (1997). Mineral binding capacity of dephytinized insoluble fiber from extruded wheat, oat and rice brans. Plant Foods Hum. Nutr..

[59] Singh S., Gamlath S., Wakeling L. (2007). Nutritional aspects of food extrusion: A review. Int. J. Food Sci. Technol..

[60] Irungu F.G., Mutungi C.M., Faraj A.K., Affognon H., Tanga C., Ekesi S., Nakimbugwe D., Fiaboe K.K.M. (2018). Minerals content of extruded fish feeds containing cricket (*Acheta domesticus*) and black soldier fly larvae (*Hermetia illucens*) fractions. Int. Aquat. Res..

[61] Vakula A., Pavlić B., Pezo L., Tepić Horecki A., Daničić T., Raičević L., Ljubojević M., Šumić Z. (2020). Vacuum drying of sweet cherry: Artificial neural networks approach in process optimization. J. Food Process. Preserv..

[62] Pandiselvam R., Manikantan M.R., Sunoj S., Sreejith S., Beegum S. (2019). Modeling of coconut milk residue incorporated rice-corn extrudates properties using multiple linear regression and artificial neural network. J. Food Process Eng..

[63] Pandey S., Kumar A., Rao P.S. (2021). Optimization, modeling, and characterization study for the physicochemical properties of raw banana and defatted soy composite extrudates. Food Chem..

[64] Kothakota A., Pandiselvam R., Siliveru K., Pandey J.P., Sagarika N., Srinivas C.H.S., Kumar A., Singh A., Prakash S.D. (2021). Modeling and optimization of process parameters for nutritional enhancement in enzymatic milled rice by multiple linear regression (MLR) and artificial neural network (ANN). Foods.

